# Development of Lipomer Nanoparticles for the Enhancement of *Drug* Release, Anti-Microbial Activity and Bioavailability of Delafloxacin

**DOI:** 10.3390/pharmaceutics12030252

**Published:** 2020-03-11

**Authors:** Md. Khalid Anwer, Muzaffar Iqbal, Magdy M. Muharram, Muqtader Mohammad, Essam Ezzeldin, Mohammed F. Aldawsari, Ahmed Alalaiwe, Faisal Imam

**Affiliations:** 1 Department of Pharmaceutics, College of Pharmacy, Prince Sattam Bin Abdulaziz University, Al-kharj 11942, Saudi Arabia; m.anwer@psau.edu.sa (M.K.A.); m.moharm@psau.edu.sa (M.M.M.); mo.ahmed@psau.edu.sa (M.M.); moh.aldawsari@psau.edu.sa (M.F.A.); a.alalaiwe@psau.edu.sa (A.A.); 2 Department of Pharmaceutical Chemistry, College of Pharmacy, King Saud University, PO Box 2457, Riyadh 11451, Saudi Arabia; esali@ksu.edu.sa; 3 Bioavailability Unit, Central Laboratory, College of Pharmacy, King Saud University, PO Box 2457, Riyadh 11451, Saudi Arabia; 4 Department of Microbiology, College of Science, Al-Azhar University, Nasr City, Cairo 11884, Egypt; 5 Department of Pharmacology and Toxicology, College of Pharmacy, King Saud University, Riyadh 11451, Saudi Arabia; fimam@ksu.edu.sa

**Keywords:** delafloxacin, LIPOMER, drug release, antimicrobial activity, pharmacokinetic

## Abstract

Delafloxacin (DFL) is a novel potent and broad-spectrum fluoroquinolone group of antibiotics effective against both Gram-positive and negative aerobic and anaerobic bacteria. In this study, DFL-loaded stearic acid (lipid) chitosan (polymer) hybrid nanoparticles (L-P-NPs) have been developed by single-emulsion-solvent evaporation technique. The mean particle size and polydispersity index (PDI) of optimized DFL-loaded L-P-NPs (F1-F3) were measured in the range of 299–368 nm and 0.215–0.269, respectively. The drug encapsulation efficiency (EE%) and loading capacity (LC%) of DFL-loaded L-P-NPs (F1-F3) were measured in the range of 64.9–80.4% and 1.7–3.8%, respectively. A sustained release of DFL was observed from optimized DFL-loaded L-P-NPs (F3). Minimum inhibitory concentration (MIC) values of the DFL-loaded L-P-NPs (F3) appeared typically to be four-fold lower than those of delafloxacin in the case of Gram-positive strains and was 2-4-fold more potent than those of delafloxacin against Gram-negative strains. The pharmacokinetic study in rats confirmed that the bioavailability (both rate and extent of absorption) of DFL-loaded L-P-NPs was significantly higher (2.3-fold) than the delafloxacin normal suspension. These results concluded that the newly optimized DFL-loaded L-P-NPs were more potent against both Gram-positive and negative strains of bacteria and highly bioavailable in comparison to delafloxacin normal suspension.

## 1. Introduction

Acute bacterial skin and skin structure infections (ABSSSIs) and community-acquired pneumonia (CAP) are considered as the most common type of infection which require hospitalization, and are the leading cause of mortality and morbidity worldwide [1,2,3]. Delafloxacin (DFL) is a novel fourth-generation fluoroquinolone group of antibiotics approved for treatment in adults for ABSSSI and CAP [4,5]. Unlike other fluoroquinolones, there is the absence of a basic group next to the fluorinated ring in the chemical structure of DFL. This change results in a weak acid property, and therefore, DFL usually exists in a deprotonated form at a neutral pH with a pKa value of 5.4, providing enhanced antibacterial potency in an acidic environment and reducing the minimum inhibitory concentrations (MICs). These properties also lead to increased accumulation and better distribution characteristics into infected target tissues than other marketed fluoroquinolones [6,7,8]. Moreover, the enzyme-inhibiting effects of DFL against DNA gyrase and topoisomerase IV is more balanced in comparison to other fluoroquinolones. Therefore, DFL exhibits very low frequencies of spontaneous mutation in vitro as an equipotent enzyme inhibition limit resistance [9,10]. DFL possesses potent and broad-spectrum antibiotic activities against both Gram-positive and negative aerobic and anaerobic bacteria, including bactericidal effects against methicillin-resistant *Staphylococcus aureus* (MRSA) [6].

DFL shows rapid absorption after oral administration and peak plasma concentration (C_max_), usually achieved within 1–2.5 h in healthy volunteers. It is widely distributed to body fluids with the volume of distribution at a steady state (V_SS_) in the range of 34–41 L and high plasma protein binding of 83–84% [11]. In compared with other flouroquinolones, DFL has low absolute bioavailability (58.8%), which may be due to its poor solubility (≈0.06 mg/mL in water). Therefore, the approved IV formulation of DFL contains sulfobutylether-β-cyclodextrin (SBECD) to enhance its solubility and stability [12]. The recommended oral dose of DFL is 450 mg, which is administered every 12 h daily. Frequent administration of conventional DFL formulation might be needed due to its low bioavailability and rapid metabolism with a mean half-life of ≤2.5 h after administration [13,14,15]. However, frequent administration of antibiotics is usually discouraging for patient compliance reasons, and it may lead to resistance, especially for CA-RTIs treatment [16].

Liposomes are believed to be an excellent biocompatible vesicular system with similarities to the biological membrane. The main concern to vesicular systems is stability, low drug encapsulation and burst drug release. However, polymer-based nanoparticles exhibit better stability over liposomes and also show sustained drug release for extended periods of time. Polymeric nanoparticles are synthesized by using synthetic (e.g., PLGA) and natural (e.g., chitosan) polymers. The use of organic solvents during the synthesis of polymeric nanoparticles limits their application [17,18,19]. The limitations of both liposomes and polymeric nanoparticles can be overcome by the synthesis of lipid polymer hybrid nanoparticles (L-P-NPs), which possess both lipid and polymeric carriers. L-P-NPs have received increasing interest in recent years, due to their superior characteristics and advantages over biopolymer-based colloidal nanoparticles [20,21]. L-P-NPs are effective in encapsulating the hydrophobic molecules with a higher drug payload than biopolymer-based nanoparticles due to their nano-range size and large surface areas. In addition, they improve drug stability and have the ability to improve the oral bioavailability of poorly water-soluble drugs [22]. Chitosan is a natural cationic polysaccharide obtained by chitin deacetylation. Due to its unique characteristics, such as nontoxicity, biocompatibility and biodegradability, as well as its favorable muco-adhesiveness and bio-membrane permeability, chitosan-coated solid-lipid nanoparticles have also been recently reported to effectively promote the in vivo absorption of poorly soluble drugs [19,23]. Recently, lipid-chitosan hybrid nanoparticles (LIPOMER nanoparticles) have been used for the oral delivery of some poorly soluble drugs [19,24,25]. In addition, the role of lipid–polymer hybrid nanoparticles in encapsulating antibiotics, e.g., chitosan-coated lipid nanoparticles loaded with rifampicin, have been evaluated for the better management of tuberculosis [26,27]. Therefore, the aim of this study was the development and optimization of novel DFL-loaded stearic acid (lipid) chitosan (polymer) hybrid nanoparticles (L-P-NPs) and their in-vitro/in vivo characterizations.

## 2. Materials and Methods

### 2.1. Chemicals and Reagents

DFL was purchased from Beijing Mesochem Technology (Beijing, China). Stearic acid, chitosan and pluronic 127 were purchased from Sigma Aldrich (St. Louis, MO, USA). All chemicals and solvents used in this study were analytical/HPLC grade. Ultra-pure water was collected from the Milli-Q water purifier unit (Millipore, Darmstadt, Germany) and was used for aqueous solution preparation.

### 2.2. Preparation of Lipid-Polymer Hybrid Nanoparticles

DFL-loaded L-P-NPs were prepared by single-emulsion-solvent evaporation technique [25]. Briefly, DFL (10 mg) was dissolved in 2 mL of prepared lipid (stearic acid) solution in ethyl acetate, and this lipid solution was further emulsified with 10 mL of chitosan solution (1 mg/mL) in 1% *w/v* acetic acid solution containing pluronic 127 surfactant (50 mg) under probe sonication (Ultrasonic processor, gx-130, Berlin, Germany) for 3 min at 60% voltage efficiency at a temperature of 25 °C (Table 1). The volatile organic solvent was evaporated by magnetic stirrer at 40 °C overnight. The DFL-loaded L-P-NPs were separated from the bulk aqueous solution by high-speed centrifugation (12,000 rpm) for 30 min, subsequently washed three times with cold distilled water and, finally, freeze-dried (Millirock Technology, Kingston, NY, USA).

### 2.3. Particle Characterization of DFL Loaded L-P-NPs

Prepared L-P-NPs were evaluated for particle size, polydispersity index (PDI) and zeta potential (ZP). The mean size and PDI of L-P-NPs (F1-F3) were measured using Malvern zeta sizer (ZEN-3600, Malvern Instruments Ltd., Holtsville, NY, USA) at 25 ± 2 °C, and dynamic light scattering (DLS) was set 90° [28]. The freshly prepared DFL-loaded L-P-NPs dispersion was diluted (100 times) with double-distilled water and sonicated for 10 min. The diluted samples (3 mL) were taken in disposable plastic cuvette and measured in size and PDI in triplicate. The diluted samples were transferred into glass electrodes in place of a glass cuvette for ZP measurement.

### 2.4. Measurement of Drug Encapsulation Efficiency

An indirect method was followed for the measurement of the percent encapsulation efficiency (EE%) and drug-loading capacity (LC%) of DFL-loaded L-P-NPs (F1-F3). Freshly prepared dispersion of nanoparticles was centrifuged (Hermle Labortechnik, Z216MK, Wehingen, Germany) at 15,000 rpm for 10 min at −4 °C to get supernatant. The supernatant was diluted appropriately, and the drug was quantified by UV spectroscopy at 290 nm. The EE% and DL% were calculated using the following equations [22]:

EE% = [(D_total_− D_free_)]/D_total_] × 100

LC% = [D_entrapped_/W_nanoparticle_] × 100

where D_total_ = amount of DFL added initially in NPs, D_free_ = DFL in supernatant, D_entrapped_ = DFL entrapped in NPs, and W_nanoparticle_ = total weight of NPs.

### 2.5. Differential Scanning Calorimetry (DSC) Studies

Thermal behaviors of pure-drug DFL and DFL in L-P-NPs (F1-F3) were studied using a DSC thermal analyzer (Scinco, DSC N-650, Seoul, Korea). Accurately weighed samples were crimped in an aluminum pan by applying pressure and heated over a temperature range of 50–300 °C at a constant heating rate of 10 °C/min. The thermal samples were purged with an inert nitrogen gas with a flow rate of 15–20 mL/min [23].

### 2.6. Fourier Transform Infrared Spectroscopy (FTIR)

FTIR spectra of pure DFL and DFL-loaded L-P-NPs (F1-F3) were recorded using the instrument FTIR spectrometer (Jasco FTIR spectrophotometer, Tokyo, Japan). Each sample was grinded with potassium bromide (10:100), and a transparent film was used by applying pressure. The spectra were recorded in the wavelength range of 4000 to 400 cm^−1^, and peaks were interpreted with the help of IR software [29].

### 2.7. In-Vitro Release Studies

A comparative release study of pure DFL and optimized DFL-loaded L-P-NPs (F3) was performed using a dialysis bag (Molecular weight cut off, 12 kDa) [30]. Briefly, pure drug DFL and F3 powder were dispersed in a dialysis bag containing 5 mL phosphate buffer (pH 7.4), and the bags were further dipped into a beaker containing 25 mL phosphate buffer at a temperature of 37 ± 1 °C with constant shaking at 100 rpm in a biological shaker (LabTech, LBS-030S, Kyonggi, Korea). At predetermined time intervals, 0.5 mL of samples were withdrawn and analyzed at 290 nm by UV spectroscopy (Jasco UV spectrophotometer V-630, Tokyo, Japan).

### 2.8. Morphology

The surface morphology and approximate size of the optimized DFL-loaded L-P-NPs (F3) were examined under transmission electron microscopy (TEM; JEOL JEM-1010, Tokyo, Japan). The DFL-loaded L-P-NPs were vortexed for 3 min after dilution with Milli-Q water. A drop of suspended DFL-loaded L-P-NP was pipped out on parafilm, and then the slide of the TEM grid was placed on the drop and left for 15 min. The grid was then removed and placed on tissue paper with the slide for 45 min for drying, then scanned for imaging.

### 2.9. In-Vitro Antimicrobial Activity

#### 2.9.1. Bacterial Isolates

For this study, seventeen type strains: *Staphylococcus aureus* (ATCC 29737), *S. aureus* (NCTC 6571), *Streptococcus pyogenes* (ATCC 12344), *S. pneumonia* (ATCC 10015), *S. agalactiae* (ATCC12386)*, Enterococcus faecalis* (ATCC 19433), *E. faecalis* (ATCC 49532), *Salmonella typhimurium* (ATCC 14028), *Pseudomonas aeruginosa* (ATCC 10145), *Staphylococcus haemolyticus* (ATCC 29970), *Proteus mirabilis* (NCIMB 13283)*, Enterobacter Aerogenes* (ATCC13048)*, E. aerogenes* (NCIMB 10102), *Bacillus subtilis* (ATCC 11774), *Klebsiella pneumonia* (ATCC 9633), *Bacillus cereus* (ATCC 10876) and *Staphylococcus epidermidis* (ATCC 12228) were examined. In addition, 10 clinical isolates of *S. aureus, S. typhimurium, E. coli*, *S. pneumonia* and *K. pneumonia* were also used. To verify the accuracy of the susceptibility results, strains of *Escherichia coli* (ATCC 25922), *Pseudomonas aeruginosa* (ATCC 27853), *Staphylococcus aureus* (ATCC 29213) and *Enterococcus faecalis* (ATCC 29212) were used as control organisms.

#### 2.9.2. Culture Conditions and Media

Strains were cultured in Mueller Hinton and stored as recommended by the manufacturer. Cultures were incubated for 18 ± 2 h at 36 ± 1 °C while streptococcal strains were incubated in Mueller-Hinton agar with 5% sheep blood under microaerobic conditions at 36 ± 1 °C for 24 h in a CO_2_ incubator.

#### 2.9.3. McFarland Standard Preparation and Inoculum Size

McFarland number 0.5 standard was made by mixing of 9.95 mL (1% H_2_SO_4_) with 0.05 mL of 1.175% barium chloride dihydrate (BaCl_2_·2H_2_O) in order to estimate bacterial density [31,32]. Preparation was stored in an airtight bottle and used for comparison of bacterial suspension whenever required. Fresh pure cultures were used for the preparation of the inoculum broth dilution of 5 × 10^5^ colony-forming units (CFU) mL^−1^.

#### 2.9.4. Minimum Inhibitory Concentration (MIC)

MICs were assessed by the agar dilution method [33,34] as recommended by the Clinical and Laboratory Standards Institute [35]. MIC values were determined and compared to activities of levofloxacin as a reference standard agent. Following standardized guidelines, bacteria were seeded in a Mueller Hinton agar medium, which was supplemented with different concentrations of DFL and DFL-loaded L-P-NPs (F3). The colony forming unit (CFU) was then counted after 24 h of incubation.

### 2.10. Bio-Analytical Methods

Our recently published UPLC-MS/MS method [36] was used for the quantitative analysis of DFL in rat plasma samples. All plasma samples were kept frozen at −80° before analysis. The method was linear in the concentration range of 3.5–5000 ng·mL^−1^. The chromatographic separation of DFL and internal standard (rivaroxaban) were performed on UPLC C_18_ columns using a mobile phase composition of acetonitrile with 0.1% formic acid and 10 mM ammonium acetate (60:40, *v/v*) at a 0.3 mL min^−1^ flow rate. The electrospray ionization in positive mode was used for triple quadrupole detection. Quantitation was accomplished with multiple reaction monitoring with parent-to-daughter ion transitions of 441.14 → 379.09 for DFL and 436.89 → 144.87 for the internal standard. The sample extractions were performed by the liquid extraction method using ethyl acetate as an extracting agent. The intra and inter-day precision (% RSD) of the method was ≤10.5, whereas the accuracy was within the range of −13.2% to 11.2% (% RE), respectively.

### 2.11. Pharmacokinetic Study in Rats

The comparative bioavailability of newly developed DFL-loaded L-P-NPs (F3) against normal suspension were evaluated in male Wistar albino rats. Twelve animals weighing between 300–350 g were received from the Animal Care Centre, College of Pharmacy, King Saud University, Riyadh, Saudi Arabia. The experimental protocol was approved by the Research Ethics Committee (Reference no. KSU-SE-19-27), and the experiment was carried out according to the guidelines of the Animal Care and Use Committee, King Saud University. Animals were divided into two groups (*n* = 6 each) and were administered DFL normal suspension and DFL-loaded L-P-NPs (F3) after overnight fasting (20 mg Kg^−1^). Blood samples were withdrawn at different time intervals (pre-dose, 0.5, 1, 1.5, 2, 3, 5, 8, 12 and 24 h). After samples were withdrawn, all blood sample were centrifuged at 5000× *g* for 10 min, and plasma were separated and stored frozen at 80 ± 10 °C until further UPLC-MS/MS analysis.

The pharmacokinetic parameters were calculated using WinNonlin software (Pharsight Co., Mountain View, CA, USA), and all values have been expressed as means ± standard deviation (SD). The noncompartmental pharmacokinetic model was used to calculate C_max_ and the time to reach maximum concentration (T_max_), AUC from 0 to t (AUC_0–24_) and 0-inf (AUC_0–inf_), elimination rate constant (kz), half-life (T½) and mean residence time (MRT).

## 3. Results and Discussion

### 3.1. Particle Characterization of DFL-Loaded L-P-NPs

The mean particle size and PDI of DFL-loaded L-P-NPs (F1-F3) were measured in the range of 299–368 nm and 0.215–0.269, respectively. The results are presented in Table 2. The lowest and largest size of the NPs were measured for F1 (299 nm) and F3 (368 nm), respectively. The results suggest that there were larger NPs with an increase in the amount of lipid in the formulation. The PDI values of F1, F2 and F3 was measured as 0.269, 0.230 and 0.215, respectively. The PDI values less than ≤0.3 are considered monodispersed particles. The ZP around ±30 mV are considered to be stable colloidal dispersions irrespective of charge (+/−), with the magnitude of charge considered accountable for stability. The ZP values of F1, F2 and F3 were found as +27.8, +20.1 and +19.2 mV, respectively. The surface charge of the lipid-chitosan hybrid nanoparticles remained positive due to the electrostatic interaction of lipid and chitosan in the formulation. The positive values of ZP were found due to the dominance of positive charges of chitosans over the negative charges of lipids (stearic acid) [23]. It was observed from the results a slight decrease in ZP with an increase in the amount of lipids (Table 2).

### 3.2. Measurement of Drug Encapsulation Efficiency

The encapsulation efficiencies of DFL-loaded L-P-NPs (F1-F3) were assessed in terms of EE% and LC%. The entrapment efficiency gives an idea of the entrapped drug, and the drug loading capacity is about the content of drugs in the nanoparticles. The EE% and LC% of DFL in L-P-NPs (F1-F3) were measured in the ranges of 64.9–80.4% and 1.7–3.8%, respectively (Table 2). It was observed from the results that increases in lipid concentrations lead to an increase in the encapsulation efficiency of DFL, probably due to resistance in the diffusion of the drug. Among the three polymer hybrid lipid NPs, the DFL-loaded L-P-NPs (F3) with compositions of SA (400 mg), CS (10 mg), pluronic 127 (50 mg) and 10 mg of DFL were found optimally with the particle size (368 nm), PDI (0.215), ZP (+19.2 mV), EE% (8.4%) and LC% (1.7%). The optimized DFL-loaded L-P-NPs (F3) were further subjected to in vitro release studies, antimicrobial assay and pharmacokinetic studies.

### 3.3. DSC Studies

**Figure 1:**
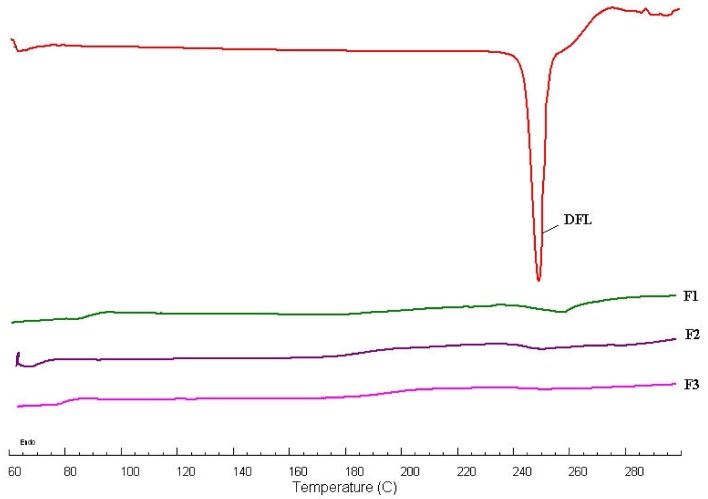
A comparative differential scanning calorimetry (DSC) spectrum of pure delafloxacin (DFL) and DFL-loaded stearic acid (lipid) chitosan (polymer) hybrid nanoparticles (L-P-NPs) (F1-F3).

DSC spectra of pure DFL and DFL in L-P-NPs (F1-F3) were scanned from 50–300 °C as shown Figure 1. A sharp endothermic peak of pure DFL at 249.32 °C evidenced its melting temperature, which was found approximately the same as reported in the literature [37]. As we can see from the DSC spectra (Figure 1), the peak of the drug (DFL) completely disappeared in DFL in L-P-NPs (F1-F3). It might be due to the encapsulation of DFL inside the lipid-polymer matrix.

### 3.4. FTIR Analysis

**Figure 2:**
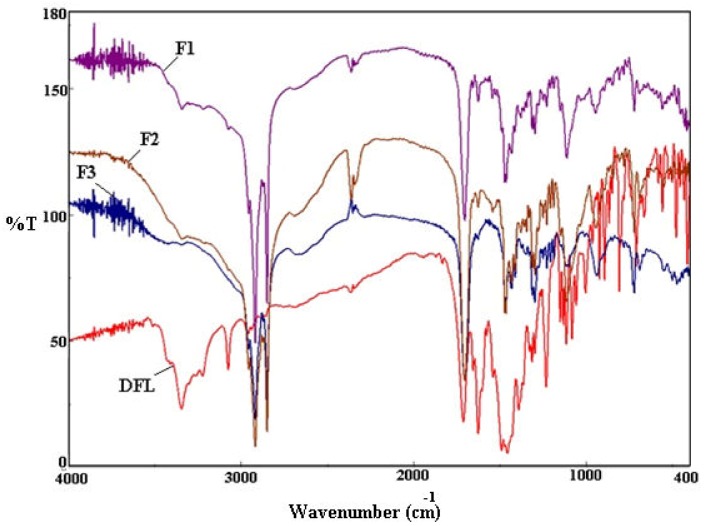
A comparative Fourier transform infrared (FTIR) spectra of pure DFL and DFL-loaded L-P-NPs (F1-F3).

The compatibility studies of the polymer (CS), lipid (SA) and drug (DFL) in nanoparticles (F1-F3) were carried out by the FTIR analysis. The comparative FTIR spectra of DFL and DFL-loaded L-P-NPs (F1-F3) were recorded for the identification and characterization of the drug (Figure 2). The major peaks of the initial DFL were assigned corresponding to the functional groups: –O–H str (3345.89 cm^−1^), –C–H str (3075.94 cm^−1^), ketone –C=O str (1715.37 cm^−1^), carboxylic –C=O str (1623.77 cm^−1^), C–F str (1114.65 cm^−1^) and C–Cl str (840.63 cm^−1^) with confirmed drug purity [31], and a reduction in the intensity of FTIR peaks was observed in the absorption spectra of DFL-loaded L-P-NPs (F1-F3) corresponding to the drug in the region of 400–1600 cm^−1^, which resulted from the overlapping of the CS, SA and DFL.

### 3.5. In-Vitro Release Studies

**Figure 3:**
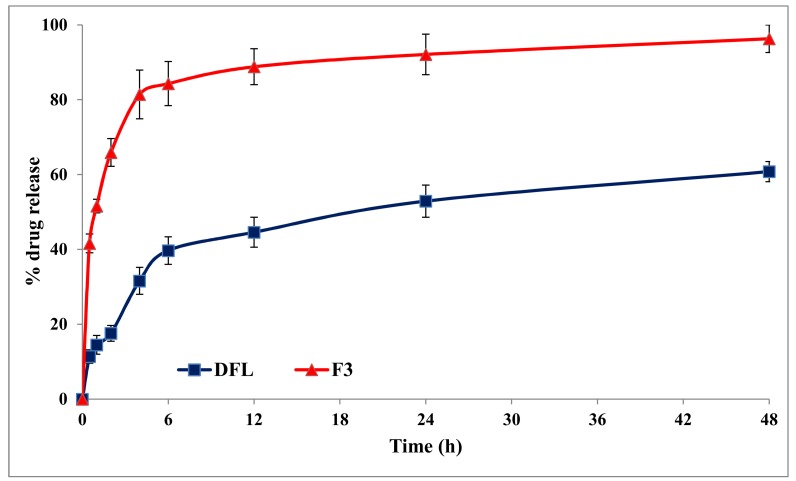
A comparative release profile of pure DFL and DFL-loaded L-P-NPs (F3).

In-vitro release studies were performed to know the pattern and mechanism of DFL from lipid polymer hybrid nanoparticles. A biphasic release pattern was observed from DFL-loaded L-P-NPs (F3), as shown in Figure 3. In the first phase, a burst release of the drug was observed at 4 h (81.4%), probably due to the surface-adsorbed drug and easy diffusion of the media into the nanoparticles. A sustained release of the drug was observed after 4 h of the study until 48 h in the second phase; it may be due to the lipid and polymer of the NPs, and subsequent immobilization may contribute to the slow release of the drug. The sustained release of the drug ultimately enhanced the bioavailability and, hence, reduced the dose and dosing frequency [38].

### 3.6. Morphology

**Figure 4:**
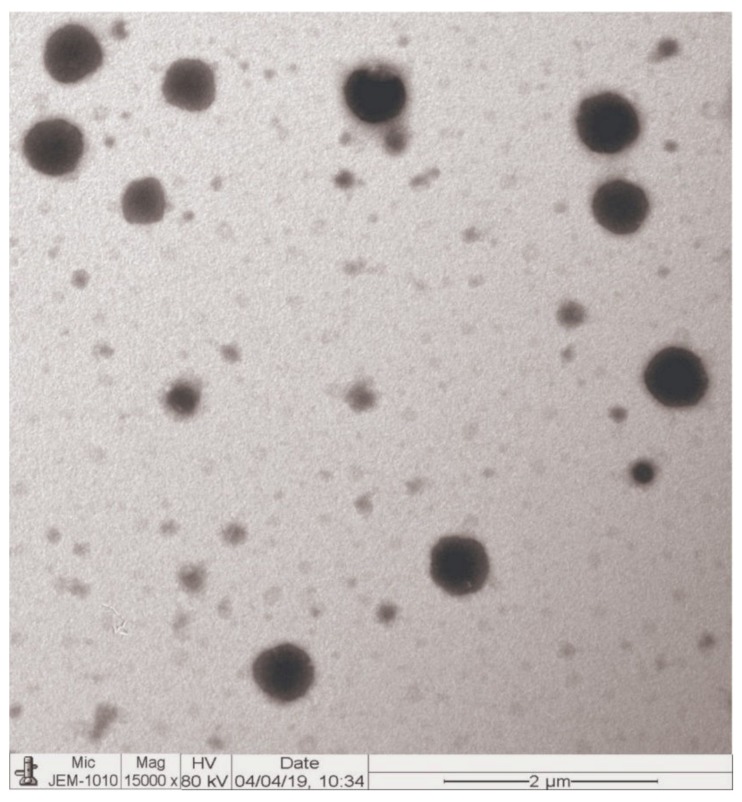
Transmission electron microscopy (TEM) images of optimized DFL-loaded L-P-NPs (F3). MIC: minimum inhibitory concentration.

TEM images of the DFL-loaded L-P-NPs (F3) are shown in Figure 4. The images of the optimized formulation (F3) showed that the prepared nanoparticles were spherical in shape, having a rough surface, and no visible aggregation of the particles was observed. The size of the F3 NPs were observed approximately the same as measured by the DLS method.

### 3.7. In Vitro Antibacterial Activity

Data presented in Table 3 summarizes the antibacterial activity of DFL and DFL-loaded L-P-NPs (F3) by MIC against some selected strains of Gram-positive and Gram-negative bacteria. Both activities were compared with levofloxacin as a reference standard agent. In the case of Gram-positive strains, MIC values were in the range of 0.0078–0.250 µg/mL and 0.0312–1.0 µg/mL with DFL and levofloxacin, respectively. However, the new DFL-loaded L-P-NPs (F3) demonstrated a potent activity against Gram-positive strains, with MIC values ranged between 0.0039 µg/mL and 0.0625 µg/mL. These results confirmed that the MIC values of the DFL-loaded L-P-NPs (F3) appeared typically to be four-fold lower than those of DFL in the case of *E. faecalis* ATCC 49,532 and two-fold lower with *S. epidermidis* ATCC 12228. Noteworthy, DFL-loaded L-P-NPs (F3) showed MIC results with Gram-negative strains with a maximal value of 0.250 µg/mL in the case of *P. mirabilis* NCIMB 13,283 and a minimal value of 0.0156 µg/mL in the case of *H. influenza* ATCC 10211. Based on the MIC data, DFL-loaded L-P-NPs (F3) were two-to-four-fold more potent than those of DFL and more potent than levofloxacin by ten-fold, as shown with *P. mirabilis* NCIMB 13,283, where the MIC value decreased from 2.0 µg/mL to 0.250 µg/mL. Higher potency of DFL-loaded L-P-NPs nanoparticles might be due to the unique structure of DFL with its nano-range size and large surface area.

### 3.8. Pharmacokinetic Studies

**Figure 5:**
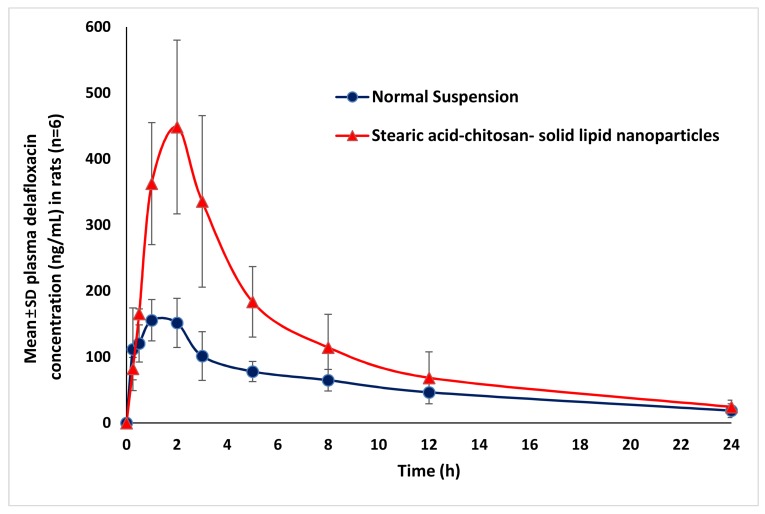
Comparative pharmacokinetic profile of DFL normal suspension and DFL loaded L-P-NPs (F3).

The comparative pharmacokinetic results obtained after an oral administration of 20 mg kg^−1^ of DFL-loaded L-P-NPs (F3) and DFL normal suspension are summarized in Table 4. As evident, both the rate and extent of absorption of DFL-loaded L-P-NPs (F3) were found to be significant C_max_ (*p* < 0.01), AUC_last_ and AUC_tot_ (*p* < 0.05) higher than the normal suspension of DFL. This results to the bioavailability of DFL-loaded L-P-NPs (F3) increased 2.3-fold in compared to the normal suspension of DFL, which proved the high circulation property of the nano-formulation in the circulatory system. Higher bioavailability of DFL-loaded L-P-NPs may be produced due to their ability to encapsulate the hydrophobic molecules with higher drug payloads and the improved drug stability of poorly water-soluble drugs like DFL. It has also been reported that the maintaining of the structural integrity of the nanoparticles is a critical factor for effective oral absorption of the drug to the site of action. Herein, a preparation of the hybrid form of lipid-polymer nanoparticles provides better structural integrity for nanocarriers [24]. Similar results have been reported in a previous study, where the bioavailability of enoxaparin-loaded L-P-NPs was 4.5-fold higher than an enoxaparin solution [25]. However, there are no significant changes in the half-life, elimination rate constant and mean resident time between these two formulations. These pharmacokinetic results are correlated with in vitro antibacterial activity results, where the potency of DFL-loaded L-P-NPs (F3) were found to be considerably higher than DFL against both Gram-positive and negative strains. Moreover, the results of the in vitro release profiles of DFL-loaded L-P-NPs (F3) were also comparable with our pharmacokinetic results. Overall, the bioavailability of DFL was vastly improved with the lipid-polymer hybrid nanoparticle system. The mean plasma concentration profiles of the DFL normal suspension and DFL-loaded L-P-NPs (F3) after an oral administration of 20 mg kg^−1^ are shown in Figure 5.

## 4. Conclusions

In this investigation, DFL-loaded L-P-NPs (F3) were formulated and optimized with the intention to enhance their bioavailability and antibacterial activity. An in vitro drug release study showed a biphasic release pattern of the optimized formulation of DFL-loaded L-P-NPs. MIC values of the DFL-loaded L-P-NPs (F3) appeared typically to be four-fold lower than those of DFL in the case of Gram-positive strains and was 2-4-fold more potent than those of DFL against Gram-negative strains. The pharmacokinetic study in rats confirms that the bioavailability of DFL-loaded L-P-NPs was significantly higher (2.3-fold) than the DFL normal suspension. Hence, it is concluded that DFL-loaded L-P-NPs promise a better therapeutic efficacy and could be a choice of replacement for the conventional formulation, with benefits of low dose requirements and better patient compliance.

Future research will involve the possibility to carry out in vitro activity for several days to confirm the stability of activity and in vivo antibacterial studies in experimental animals.

## Figures and Tables

**Table 1:** Composition of delafloxacin (DFL)-loaded stearic acid (lipid) chitosan (polymer) hybrid nanoparticles (L-P-NPs).

Formulae	DFL (mg)	Stearic Acid (mg)	Chitosan (mg)	Pluronic 127 (mg)
F1	10	100	10	50
F2	10	200	10	50
F3	10	400	10	50

**Table 2:** Evaluations of DFL-loaded L-P-NPs (mean ± SD, *n* = 3). PDI: polydispersity index, ZP: zeta potential, EE%: drug encapsulation efficiency and LC%: loading capacity.

Formulae	Stearic Acid (mg)	Size (nm)	PDI	ZP (mV)	EE%	LC%
F1	100	299 ± 2.7	0.269 ± 0.042	+27.8 ± 3.2	64.9 ± 2.3	3.8 ± 0.3
F2	200	345 ± 3.9	0.230 ± 0.018	+20.1 ± 1.5	76.1 ± 1.6	2.8 ± 0.5
F3	400	368 ± 5.2	0.215 ± 0.015	+19.2 ± 1.4	80.4 ± 3.1	1.7 ± 0.1

**Table 3:** Minimum inhibitory concentrations (MICs) (µg/mL) of DFL and its DFL-loaded L-P-NPs (F3) against bacterial pathogens compared with levofloxacin.

Bacterial Strain	DFL	F3	Levofloxacin
*Escherichia coil* (ATCC25922)	0.1250	0.0312	1.0
*Escherichia coil* (ATCC 11229)	0.1250	0.0625	1.0
*Escherichia coil* (Clinical isolate)	0.50	0.1250	1.5
*Klebsiella pneumonia* (ATCC 9633)	0.1250	0.0625	0.50
*Klebsiella pneumonia* (Clinical isolate)	0.0625	0.0312	0.125
*Pseudomonas aeruginosa* (ATCC 27853)	0.50	0.125	2.5
*Pseudomonas aeruginosa* (ATCC 10145)	0.50	0.125	2.0
*Escherichia aerogenes* (ATCC13048)	0.50	0.125	1.0
*Escherichia aerogenes* (NCIMB 10102)	1.0	0.250	1.5
*Haemophilus influenza* (ATCC 10211)	0.0312	0.0156	0.125
*Pseudomonas mirabilis* (NCIMB13283)	1.0	0.250	2.0
*Salmonella typhimurium* (ATCC 14028)	0.1250	0.0312	0.50
*Salmonella typhimurium* (Clinical isolate)	0.1250	0.0312	0.50
*Salmonella aureus* (ATCC 29213)	0.0156	0.0078	0.50
*Salmonella aureus* (ATCC 25923)	0.1250	0.0625	0.50
*Salmonella aureus* (NCTC 6571)	0.0156	0.0078	0.50
*Salmonella aureus* (ATCC 29737)	0.0156	0.0078	0.250
*Salmonella aureus* (Clinical isolate)	0.0156	0.0078	0.50
*Salmonella epidermidis* (ATCC 12228)	0.0078	0.0039	0.0312
*Salmonella pneumonia* (ATCC 49619)	0.0156	0.0078	0.50
*Salmonella haemolyticus* (ATCC 29970)	0.250	0.0625	1.0
*Bacillus subtilis* (ATCC 11774)	0.0156	0.0039	0.0312
*Bacillus cereus* (ATCC 10876)	0.0312	0.0078	0.0625
*Escherichia faecalis* (ATCC 29212)	0.0625	0.0312	0.50
*Escherichia faecalis* (ATCC 19433)	0.1250	0.0312	0.50
*Escherichia faecalis* (ATCC 49532)	0.250	0.0625	1.0

**Table 4:** Comparative pharmacokinetic profile of DFL-loaded L-P-NPs (F3) vs. normal suspension.

Parameters	Normal Suspension (Mean ± SD, *n* = 6)	DFL-Loaded L-P-NPs (F3) (Mean ± SD, *n* = 6)
*C_max_ (ng/mL)*	231 ± 67	597 ± 228 ^**^
*T_max_ (h)*	1	2
*AUC_last_ (ng/h/mL)*	1618 ± 301	3717 ± 1600 ^*^
*AUC_tot_ (ng/h/mL)*	2084 ± 106	3895 ± 1508 ^*^
*K_el_ (h)*	0.087 ± 0.038	0.130 ± 0.038
*T_1/2_ (h)*	6.17 ± 0.27	5.70 ± 1.94
*MRT (h)*	9.72 ± 2.04	7.77 ± 2.81
*Relative Bioavailability (%)*	100	230%

C_max_ = maximum plasma concentration; T_max_ = Time to C_max_; AUC =Area under curve; K_el_ = elimination rate constant; T_1/2_ = half-life; MRT = mean residence time. * *p* ˂ 0.05 significant compared with normal suspension and ** *p* ˂ 0.01 highly significant compared with normal suspension.
